# Effect of dietary antioxidants on free radical damage in dogs and cats

**DOI:** 10.1093/jas/skae153

**Published:** 2024-06-03

**Authors:** Dennis E Jewell, Laura A Motsinger, Inke Paetau-Robinson

**Affiliations:** Department of Grain Science and Industry, Kansas State University, Manhattan, KS, USA; Hill’s Pet Nutrition, Inc., Topeka, KS, USA; Compana Pet Brands, St. Louis, MO, USA

**Keywords:** antioxidants, cat, dog, oxidative stress, vitamin C, vitamin E

## Abstract

Alpha-tocopherol (vitamin E) is an antioxidant that is largely involved in immune defense and enhancing the ability of biological systems to respond to oxidative stress. During the process of free radical scavenging, vitamin C supports the regeneration of vitamin E. Although the functions of antioxidants and their importance have been widely studied, the intricate interplay between antioxidants has yet to be fully elucidated, especially in dogs and cats. As such, the objective of the present study was to determine the effect of a combination of dietary antioxidants on DNA damage and antioxidant status in dogs and cats. Forty adult mixed-breed dogs and 40 adult domestic shorthair cats were randomly assigned to one of four treatment groups per species. Dogs and cats remained in these groups for the 84-d duration of the study. The food differed in antioxidant supplementation with the control food meeting all of the Association of American Feed Control Officials requirements for complete and balanced nutrition, including sufficient vitamin E to exceed the published minimum. The treatment diets were targeted to include either 500, 1,000, or 1,500 IU vitamin E/kg as well as 100 ppm of vitamin C and 1.5 ppm of β-carotene in the food. The effect of vitamin E supplementation level on serum vitamin E concentration, DNA damage, and total antioxidant power was evaluated. Feeding diets enriched with antioxidants resulted in an increased (*P* < 0.05) circulating vitamin E concentration, increased (*P* < 0.05) immune cell protection, reduced (*P* < 0.05) DNA damage in dogs, and an improved (*P* < 0.05) antioxidant status. Overall, these data demonstrated that feeding a dry kibble with an antioxidant blend inclusive of vitamin E, vitamin C, and β-carotene enhanced cell protection and improved antioxidant status in dogs and cats.

## Teaser

Dogs and cats fed an antioxidant blend inclusive of vitamin E, vitamin C, and β-carotene have enhanced immune health, as observed through reduced cellular breakdown and reduced signs of oxidation with increasing concentrations of the antioxidants.

## Introduction

Antioxidants support many bodily functions and are particularly involved in sustaining immunological defense. The immune system performs a delicate balancing act via control mechanisms activated by positive and negative feedback as it not only differentiates between self and foreign, but also governs the level of response to viral, bacterial, and other identified intruders. Imbalance of this system can result in an exaggerated immune response, or the sluggish and less effective response, to invasion that is typical of an immunosuppressed individual. Influenced by many factors, it is well known that aging results in a decline of the ability of this system to respond (Hall et al., 2010; Weyand and Goronzy, 2016). One source of immune system damage, especially in an aging animal, is exposure to excessive reactive oxygen species (**ROS**). The production of ROS is normal and a part of biological life and health. However, an imbalance of oxidative stress (**OS**) and antioxidants may result in increased cellular damage (Sies et al, 2022).

Endogenous antioxidant defenses do not prevent all ROS damage, making antioxidant supplementation an important adjunct to maintaining health and improving immune system responses. The assertion that vitamin E supports an immune system reaction has been well established. In fact, in a review of nutrition and immunity interactions, Klasing and Leshchinsky (2000) stated that “almost every aspect of the immune system has been shown to be altered by the dietary level of vitamin E, including resistance to infection, specific antibody responses, number of splenic plaque-forming cells, in vitro mitogenic responses of lymphocytes and phagocytic index.” These authors defended their assertions by referring to many reports of improved immunocompetence and resistance to subclinical infection resulting from enhanced vitamin E nutrition.

Certainly, vitamin E has been shown to have a positive effect on immune system response and strengthens the ability of biological systems to respond to OS, but it does not act in a vacuum, and interaction of the various antioxidants must be taken into account. Free radical scavenger antioxidants, such as vitamin E, react with and detoxify ROS, but this may result in the destruction of the antioxidant. As such, there is an advantage to ingesting a mix of dietary antioxidants, illustrated by the fact that lipoic acid can regenerate vitamin C, which can, in turn, regenerate vitamin E (Betancor, et al, 2012; Rochette, et al, 2013; Tibullo et al, 2017). The combination of vitamin E, vitamin C, and carotenoids has been suggested to be a benefit through reduced oxidative damage (Head et al, 2008; Panickar and Jewell, 2015). Although the intricate give-and-take interplay between antioxidants has been studied extensively, the interaction has not been fully elucidated, especially in dogs and cats. As such, the objective of this study was to evaluate the potential of antioxidants as a method of improving antioxidant status and cellular health in dogs and cats. The authors of the present study hypothesized that circulating vitamin E, OS, and total antioxidant power (**TAP**), would be improved by the provision of increasing concentrations of vitamin E in the diet.

## Materials and Methods

### Animals

All experimental procedures were reviewed and approved by the Hill’s Institutional Animal Care and Use Committee. Forty dogs and 40 healthy cats were fed to maintain body weight while being allowed free access to water. Dogs were beagles (average weight 12.0 kg, average daily intake 257.9 g, 30 spayed females, and 10 neutered males with an average age of 3.5 yr) and cats were domestic shorthair (average weight 3.1 kg, average daily intake 54.4 g, 32 spayed females and 8 neutered males with an average age of 4.0 yr). All dogs and cats were maintained similar to those previously described (Jewell and Jackson, 2022; Jewell et al., 2022). In short, body weight was recorded weekly and food intake was adjusted as needed to maintain body weight. Dogs and cats were assigned to treatment based on gender and age. To do this, all available pets were used and they were balanced by gender by first assigning females to their respective treatment groups and then the remaining males were assigned by age so that similar age was present across all treatment groups. Blood samples were taken after an overnight fast. Blood was collected for the Comet assay, circulating vitamin E (initial and final), TAP, and 8-hydroxy-2ʹ-deoxyguanosine at the end of the study.

### Diets

Dogs and cats were randomly assigned to one of four dietary treatment groups and fed the treatment foods for 84 d. Before day 0 dogs and cats were maintained on the control food for 2 wk. Dietary treatments consisted of foods provided as extruded kibbles: a control food meeting all of the Association of American Feed Control Officials (**AAFCO**) requirements for adult maintenance and three treatment foods with incrementally increased α-tocopherol (vitamin E) supplementation. The control diets included 291 and 123 IU vitamin E/kg, 5 and 24 ppm of vitamin C, and 0.3 and 0.1 ppm of β-carotene in canine and feline diets, respectively, as measured by analyses. The foods with increased antioxidant supplementation had an increased formulation supplement of 100 ppm of vitamin C and 1.5 ppm of β-carotene. Vitamin E was progressively increased in added amounts of 500, 1,000, and 1,500 IU vitamin E/kg in the supplemented foods. Vitamin E (α-tocopherol) and β-carotene were purchased from DSM. Vitamin C was provided in the form of a ROVIMIX STAY-C (DSM Nutritional Products, Inc., Amsterdam, the Netherlands), a stabilized Na/Ca salt of l-ascorbic acid. Food composition is described in Table 1.

**Table 1.** Concentrations of selected nutrients (as-fed basis)

	Dog				Cat			
	Treatment diet^1^							
Nutrient	Control^2^	500	1,000	1,500	Control^3^	500	1,000	1,500
Crude protein, %	20.6	21.6	20.6	20.6	31.2	32.1	31.0	31.4
Crude fat, %	15.6	15.8	15.6	15.4	15.7	15.8	15.0	15.1
Ash, %	4.2	4.1	4.2	4.1	5.0	4.9	5.0	5.6
Crude fiber, %	2.7	2.5	2.5	2.6	0.8	0.6	1.0	0.9
Moisture, %	9.0	8.4	8.4	7.8	7.2	6.0	7.3	6.9
ME, kcal/kg^4^	3,724	3,765	3,752	3,763	3,830	3,888	3,785	3,786
β-Carotene, ppm	0.26	0.47	0.65	0.58	0.82	1.15	1.16	1.26
Vitamin E, IU/kg	290.7	409.9	986.7	1,333.4	123.2	407.8	997.8	1,396.1
Vitamin C, ppm	5.15	41.2	63.0	59.0	23.8	71.9	63.7	69.0

^1^Treatment diets included the control diet (291 and 123 IU vitamin E/kg in canine and feline diets, respectively), and diets that included 500, 1,000, and 1,500 formulated IU vitamin E/kg in the diet.

^2^Ingredient composition of diet: corn, poultry byproduct meal, brewers rice, corn gluten meal, pork fat, natural flavor, dried beet pulp, soybean oil, calcium carbonate, potassium chloride, choline chloride, iodized salt, vitamins (vitamin E supplement α tocopherol acetate—concentration changed to meet formulation, l-ascorbyl-2-polyphosphate—concentration changed to meet formulation, β-carotene supplement—concentration changed to meet formulation, niacin supplement, thiamin mononitrate, vitamin A supplement, calcium pantothenate, riboflavin supplement, biotin, vitamin B12 supplement, pyridoxine hydrochloride, folic acid, vitamin D3 supplement), and minerals (ferrous sulfate, zinc oxide, copper sulfate, manganous oxide, calcium iodate, and sodium selenite).

^3^Brewers rice, poultry byproduct meal, corn gluten meal, corn, pork fat, natural flavor, soybean oil, calcium sulfate, potassium chloride, choline chloride, potassium chloride, vitamins (vitamin E supplement, α tocopherol acetate—concentration changed to meet formulation, l-ascorbyl-2-polyphosphate—concentration changed to meet formulation, β-carotene supplement—concentration changed to meet formulation, niacin supplement, thiamin mononitrate, calcium pantothenate, pyridoxine hydrochloride, vitamin A supplement, riboflavin supplement, biotin, vitamin B12 supplement, folic acid, and vitamin D3 supplement), taurine, iodized salt, and minerals (ferrous sulfate, zinc oxide, copper sulfate, manganous oxide, calcium iodate, and sodium selenite).

^4^Values are calculated using modified Atwater coefficients (Jewell and Jackson, 2023).

### Blood analyses

Blood was drawn at the beginning and end of the study and analyzed for vitamin E, TAP, the Comet assay, and 8-hydroxy-2ʹ-deoxyguanosine.

The antioxidant capacity of the sera was determined by measuring the ability of the antioxidants in the sample to reduce Cu++ to Cu+ and the antioxidant concentrations in each sample were determined by comparing to a standard curve that was developed from known concentrations of uric acid using a colorimetric microplate assay and reported as TAP (Product No. TA 02, Oxford Biomedical Research, Oxford, MI). The Comet assay is a gel electrophoresis-based method that can be used to measure DNA damage in individual eukaryotic cells from ethylenediaminetetraacetic acid treated whole blood and the authors used methods from Heaton et al. (2002) for this analysis. The DNA breakdown product, 8-hydroxy-2ʹ-deoxyguanosine, was measured in the serum through a standard methodology (spectrophotometric ELISA kit produced by Genox Corporation, Baltimore, MD).

### Statistical analyses

All statistical analyses were completed using SAS (version 9.4; SAS Institute Inc., Cary, NC, USA). ANOVA was accomplished using the MIXED procedure. This study was a completely randomized design with gender as a block. However, gender was not found to be significant as a block and was not used in the statistical evaluation. Each species was evaluated independently. Treatment was used as the main effect of the statistical model. The analysis for a linear or quadratic effect was completed using a contrast statement in the MIXED procedure. The appropriateness of using the MIXED procedure and the assumption of normality was evaluated by using the Shapiro–Wilk tests as well as by evaluating the normal probability plots. A *P* ≤ 0.05 was considered significant.

## Results

### Serum vitamin E

Analysis of serum vitamin E concentration demonstrated that both dogs and cats had increased circulating vitamin E concentrations with the addition of increased dietary vitamin E. Both species had a linear increase (*P *< 0.01) and a quadratic response (*P *< 0.05) for both change over time and final values when vitamin E level was increased in the diets (Table 2).

**Table 2.** Circulating vitamin E response to dietary supplementation

Treatment diet^1^	Vitamin E (μg/ml) dog serum			Vitamin E (μg/ml) cat serum		
	Change^2^	Initial	Final^2^	Change^2^	Initial	Final^2^
Control	3.5^c^	21.8	25.3^b^	4.7^c^	11.1	15.8^b^
500	18.9^b^	21.3	40.2^a^	10.2^b^	10.5	20.6^b^
1,000	24.6^a^	22.8	47.4^a^	24.1^a^	11.6	35.8^a^
1,500	27.2^a^	20.8	48.0^a^	21.8^a^	11.0	32.8^a^
SD	8.7	5.8	10.3	5.7	2.5	5.7

^1^Treatment diets included the control diet (291 and 123 IU vitamin E/kg in canine and feline diets, respectively), and diets that targeted 500, 1,000, and 1,500 IU vitamin E/kg in the diet.

^2^Serum vitamin E increased in a linear (*P *≤ 0.01) and quadratic (*P *≤ 0.05) fashion as the level of vitamin E supplementation in the diet increased.

^a,b,c^Means with different superscript letters in the same column are different (*P* < 0.05).

ME, metabolizable energy.

### Comet assay

Analysis of the amount of DNA remaining in the head of the cells during the Comet assay showed that increasing dietary vitamin E increased head DNA. There was a linear effect (*P* < 0.05) of increased dietary antioxidants in both dogs and cats (Table 3). With increased concentrations of supplemented vitamin E, there was an increased (*P *< 0.05) retention of DNA in the nuclei as shown by the increased percentage of head DNA in dogs and cats (Table 3).

**Table 3.** Canine and feline Comet assay at end of study

Treatment diet^1^	Dog (% head DNA)^2^	Cat (% head DNA)^2^
Control	72.0^b^	73.5^b^
500	75.8^a,b^	75.5^a,b^
1,000	76.2^a,b^	77.1^a,b^
1,500	79.1^a^	79.8^a^
SD	5.6	5.8

^1^Treatment diets included the control diet (291 and 123 IU vitamin E/kg in canine and feline diets, respectively), and diets that included 500, 1,000, and 1,500 IU vitamin E/kg in the diet.

^2^Linear effect of antioxidant supplementation.

### TAP and 8-hydroxy-2ʹ-deoxyguanosine concentrations

In cats, there was a linear increase (*P* < 0.05) in TAP with increasing dietary vitamin E and the 1,500 IU vitamin E/kg supplemented group mean was increased as compared to the control (Table 4). In dogs, there was a quadratic response in TAP with increasing dietary vitamin E with the 500 supplemented group mean being elevated over the control (Table 4). There was also a decrease (*P *< 0.05) in 8-hydroxy-2ʹ-deoxyguanosine when dogs were supplemented with increased dietary vitamin E (Table 4).

**Table 4.** The effect of antioxidant supplementation on TAP and 8-OHdG concentrations in the serum of dogs and cats

Treatment diet^1^	Dog TAP^3^	Dog 8-OHdG	Cat TAP^2^	Cat 8-OHdG^2^^,^^3^
Control	0.184^b^	36.5^b^	0.128^b^	11.2
500	0.320^a^	24.4^a,b^	0.136^a,b^	9.3
1,000	0.218^a,b^	26.9^a,b^	0.181^a,b^	7.7
1,500	0.215^a,b^	24.1^a^	0.217^a^	4.0
SD	0.093	10.0	0.076	10.9

^1^Treatment diets included the control diet (291 and 123 IU vitamin E/kg in canine and feline diets, respectively), and diets that included 500, 1,000, and 1,500 IU vitamin E/kg in the diet.

^2^Linear effect of antioxidant supplementation.

^3^Quadratic effect of antioxidant supplementation.

TAP, total antioxidant power; 8-OHdG, 8-hydroxy-2ʹ-deoxyguanosine, ng/mL.

## Discussion

Endogenous antioxidant defenses do not prevent all ROS damage, making antioxidant supplementation at concentrations much higher than the minimum National Research Council recommendations (National Research Council, 2006) an important adjunct to maintaining health. Certainly, there is good reason for the recommendation for people to eat at least two servings of fruits and three servings of vegetables daily. The NRC minimum is established based on evidence to prevent obvious nutrient deficiencies, but data resulting from increased consumption exceeding the minimum are incomplete and expose a need for further research to define more optimum dietary concentrations rather than those needed for basic survival. The consumption of a naturally available mix of antioxidants and micronutrients may provide enhanced health benefits.

The addition of dietary antioxidants, such as vitamin E, vitamin C, and β-carotene, at doses much higher than previous recommended dietary concentrations for a complete and balanced diet (National Research Council, 2006; AAFCO, 2023) is not meant to replace the antioxidants and other nutrients available naturally in food, but to supplement these for optimum health. In the present study, analysis of increasing concentrations of dietary vitamin E supplementation showed that as vitamin E supplementation increased, serum concentrations of vitamin E also increased in dogs and cats; however, a threshold was reached at supplementation of 1,000 IU vitamin E/kg to foods. Previous studies have observed increased circulating concentrations in response to increased consumption. Panickar et al. (2014) demonstrated that increasing concentrations of dietary vitamin E in the diet (293, 445, and 598 IU vitamin E/kg as-fed in canine diets and 248, 384, and 540 IU vitamin E/kg as-fed in feline diets), resulted in increased serum vitamin E concentrations in dogs and cats. In a study by Hall et al. (2010), feeding diets enriched with ≥640 IU vitamin E/kg and 130 mg vitamin C/kg food (as-fed) to geriatric beagles resulted in increased serum vitamin E concentrations. Additionally, in a study by Heaton et al. (2002), serum vitamin E concentrations were increased after 4 wk of supplementation with a blend of antioxidants inclusive of vitamin E, although the level of vitamin E that was fed was not disclosed. Overall, these data indicate that as vitamin E supplementation increases in the diet, serum concentration also increases, but there appears to be limited additional increase in circulating concentration of vitamin E over that achieved in the groups consuming 1,000 IU/kg of supplementary vitamin E in both dogs and cats.

The Comet assay is a method for determining DNA damage by measuring breaks in DNA strands. In the present study, as dietary concentrations of supplemented vitamin E increased, there was a linear increase in the retention of DNA in the nuclei, as shown by the increased percentage of head DNA in both dogs and cats. Yu and Paetau-Robinson (2006) observed similar results and demonstrated that supplementing the diet with vitamin E, vitamin C, and β-carotene for 4 wk reduces DNA damage as measured by Comet assay parameters in cats with spontaneous renal insufficiency. Heaton et al. (2002) also observed similar results and showed that dogs fed a diet that was supplemented with an antioxidant blend inclusive of vitamin E, vitamin C, β-carotene, taurine, lutein, and lycopene, had a reduction in DNA damage as measured by the Comet assay. These data indicate that DNA damage is decreased in dogs when vitamin C, β-carotene, and increasing concentrations of vitamin E are fed in the diet. In cats, further research is needed to understand what DNA benefit is present with the increased concentrations of circulating vitamin E as head DNA was increased with increasing concentration, but there was not a reduction in 8-hydroxy-2ʹ-deoxyguanosine in cats.

The oxidized derivative of deoxyguanosine, 8-hydroxy-2ʹ-deoxyguanosine, is another marker of DNA oxidation/damage. Mutations resulting from DNA oxidation may cause mitochondrial dysfunction (Hagen et al., 1997; Cottrell et al., 2000) and mitochondrial impairment may be a significant contributor to aging (Harman, 1972; Shigenaga et al., 1994). Lesions in mitochondrial deoxyribonucleic acid (**mtDNA**) are thought to be caused by increased ROS as age increases. In fact, when brain tissue from humans aged 42 to 97 yr was tested for the oxidized nucleoside 8-hydroxy-2ʹ-deoxyguanosine, a 10-fold increase in 8-hydroxy-2ʹ-deoxyguanosine was found in mtDNA as compared to nuclear DNA (Mecocci et al., 1993). People older than 70-yr-of-age have a 15-fold increase in 8-hydroxy-2ʹ-deoxyguanosine (Mecocci et al., 1993). The result of mtDNA damage may be inefficient electron transport and, hence, increased production of ROS and inability to maintain membrane potential. To assess the impact of dietary antioxidant supplementation on DNA damage, the present study evaluated 8-hydroxy-2ʹ-deoxyguanosine. In dogs, increasing dietary antioxidants resulted in decreased 8-hydroxy-2ʹ-deoxyguanosine. Similar to the results observed in the present study, Panickar et al. (2014) demonstrated that feeding aging dogs a diet that is supplemented with a blend of ingredients known to have potent antioxidant and anti-inflammatory effects (carrots, spinach, tomato pomace, carnitine, and α-lipoic acid) results in decreased presence of 8-hydroxy-2ʹ-deoxyguanosine. In another study by Jewell et al. (2000), serum alkenal concentrations, another measure of oxidative reaction, specifically lipid peroxidation, were decreased in dogs and cats that were supplemented with 445 and 540 IU vitamin E/kg food, respectively, on an as-fed basis. Yu and Paetau-Robinson (2006) demonstrated that supplementing the diet with vitamin E, vitamin C, and β-carotene for 4 wk reduced 8-hydroxy-2ʹ-deoxyguanosine in cats with spontaneous renal insufficiency. The results of the present study, in addition to results observed from previous research, indicate that increased dietary concentrations of antioxidants may decrease indicators of oxidative damage and feeding a blend of antioxidants helps prevent oxidation and subsequent DNA damage.

This study was designed to evaluate the effect of increasing dietary antioxidants on antioxidant status and cell protection. To accomplish this, the antioxidant capacity of the sera was determined by measuring the ability of the antioxidants in the sample to reduce Cu++ to Cu+ and reported as TAP. Both dogs and cats had an increase in TAP when antioxidant-supplemented groups were compared to the control groups. Results of the present study are in agreement with results observed by Dunlap et al. (2006) who demonstrated that feeding sled dogs a diet inclusive of blueberries, which are rich in flavonoids and polyphenols, increased TAP post-exercise when compared to the control group of dogs that were not fed the diet that included blueberries and were not exercised.

Many studies have been performed on the health influence of various combinations of antioxidants. A prime example is the benefit seen from supplementing vitamin E with selenium. Vitamin E and selenium given to dogs in quantities above established dietary requirements were found to stimulate the immune system (Sheffy and Schultz, 1979). A feeding trial reported by Harper et al. (2001) indicated an improved antibody response to feline calicivirus vaccination in cats consuming enhanced concentrations of vitamin E, β-carotene, taurine, and lutein when compared to cats consuming a basal food. A study by Heaton et al. (2002) compared two groups of adult dogs, one on a control food and the other on a food supplemented with vitamin E, vitamin C, β-carotene, taurine, lutein, and lycopene for 12 wk. The dogs on antioxidant supplementation had less evidence of endogenous and exogenous DNA damage and had significantly improved immune response to vaccination. Massimino et al. (2001) compared immune system response between growing Beagles on a control food with similar Beagles on food supplemented with 200 IU vitamin E, 20 mg lutein, and 20 mg β-carotene/kg food and found that the antioxidant-supplemented group had significantly improved immune response while developing higher titers in response to distemper and parvovirus vaccines. Chew et al. (2000) demonstrated the influence of dietary β-carotene in dogs and found that it enhanced measures of both humoral and cell-mediated immunity. Overall, these data as a whole confirm that antioxidants enhance the immune response in dogs and cats. This may be through the cell protection capabilities found in this study with increased immune cell integrity as shown by the Comet assay and reduced oxidative DNA breakdown as shown by the response in 8-hydroxy-2ʹ-deoxyguanosine in dogs.

In summary, these data show that there is an enhanced cell protection associated with adding increasing concentrations of dietary antioxidants. The quadratic response to increasing dietary vitamin E in some responses (e.g., circulating vitamin E concentrations) shows improvement in antioxidant status does not simply always increase with increasing supplementation. Some responses (e.g., percent head DNA) in cats and dogs also support the conclusion that optimal dietary intake may be different for each specific response variable as it improved throughout the supplemented range.

## Conclusions

These results are valuable indications of the range of vitamin E supplementation that should be considered in diets for healthy dogs and cats. Both dogs and cats benefited from increased dietary antioxidants with circulating vitamin E concentration maximized when vitamin E was provided at approximately 1,000 IU/kg in the food. Overall, these data demonstrate that dogs and cats fed an antioxidant blend inclusive of vitamin E, vitamin C, and β-carotene have improved immune health as observed through reduced signs of oxidation and decreased cellular breakdown.

## Abbreviations

AAFCO: Association of American Feed Control Officials
EDTA: ethylenediaminetetraacetic acid
mtDNA: , mitochondrial deoxyribonucleic acid
NRC: National Research Council
OS: oxidative stress
ROS: reactive oxygen species
TAP: total antioxidant power
